# Stability Trends in Mono-Metallic 3d Layered Double Hydroxides

**DOI:** 10.3390/nano12081339

**Published:** 2022-04-13

**Authors:** Saeedeh Mohammadi, Ayoub Esmailpour, Esmail Doustkhah, Mohammad Hussein Naseef Assadi

**Affiliations:** 1 Department of Physics, Shahid Rajaee Teacher Training University, Lavizan, Tehran 16788-15811, Iran; s.mohammadi@sru.ac.ir (S.M.); esmailpour@sru.ac.ir (A.E.); 2 Koç University Tüpraş Energy Center (KUTEM), Department of Chemistry, Koç University, Istanbul 34450, Turkey; 3 RIKEN Center for Emergent Matter Science (CEMS), 2-1 Hirosawa, Wako, Saitama 351-0198, Japan

**Keywords:** density functional theory, green rust, LDA + *U*, layered double hydroxides, LDH, intercalation, stability

## Abstract

Layered double hydroxides (LDHs) constitute a unique group of 2D materials that can deliver exceptional catalytic, optical, and electronic performance. However, they usually suffer from low stability compared to their oxide counterparts. Using density functional calculations, we quantitatively demonstrate the crucial impact of the intercalants (i.e., water, lactate, and carbonate) on the stability of a series of common LDHs based on Mn, Fe, and Co. We found that intercalation with the singly charged lactate results in higher stability in all these LDH compounds, compared to neutral water and doubly charged carbonate. Furthermore, we show that the dispersion effect aids the stability of these LDH compounds. This investigation reveals that certain intercalants enhance LDH stability and alter the bandgap favourably.

## 1. Introduction

By having atomically thin layers and internal nano-space intercalation, layered materials are at the frontier of nanomaterial research [1,2,3]. Among layered materials, layered double hydroxides (LDHs) [4] constitute a substantial class with a broad range of applications in the catalysis of organic transformations, wastewaters and pollutant degradation, CO_2_ capture [5,6], templating for oriented synthesis [7], photocatalysis [5,8], supercapacitance [9], membrane fabrication [10], and drug delivery [11,12]. LDHs are usually composed of divalent or trivalent metals with a general formula of $[(M_{1-x}^{\mathrm{II}}M_{x}^{\mathrm{III}}{{\left(\mathrm{OH}\right)}_{2}]}^{x+}:\left(A_{x/m}^{m-}\right)\cdot nH_{2}O;$ *x* = ~0.2–0.4, where the M^II^ and $M^{\mathrm{III}}$ can be either identical or different metallic ions. The LDH layers are positively charged and neutralised by anionic intercalants (A^m−^). Carbonate [13,14,15,16,17], nitrate [18], sulphate [13,16], and lactate [19] are some examples of the anionic intercalants in LDHs that compensate the positive charge of the LDH layers [20]. Recent studies suggest that the intercalant anions play a crucial role in determining the physicochemical properties of the final LDHs, opening the possibility of engineered LDHs for specific applications [21]. Therefore, understanding how guest anions influence the structural and electronic properties in LDHs is essential for tailoring their properties.

Carbonate, one example of an intercalating anion, has three oxygens and therefore possesses a strong hydrogen bonding capacity with the interlayer surface of an LDH, thus showing a high adsorption tendency with LDHs [14,22]. Previously, some carbonate-intercalated LDHs such as $Mg-{Al}_{\left(1/3\right)}$ LDH [6], strätlingite (Ca_2_Al(AlSi)O_2_(OH)_10_·2.25H_2_O) [16], and hydrotalcite (Mg_0.7_Al_0.3_(OH)_2_(CO_3_)_0.15_·0.63H_2_O) [22,23] were experimentally investigated and reported. Lactate as another intercalant anion is utilised in the intercalation of Fe LDH interlayers, showing a potential application in the design of catalytically active material for $H_{2}$ production [19]. Most of the presented examples from the literature are for bimetallic LDHs. Monometallic LDHs are rarely studied and investigated in detail due to their lower stability and synthesis difficulties [24]. In particular, producing and maintaining both divalent or trivalent cations of the same metal in the application medium is challenging [25]. Furthermore, the difficulty in differentiating the divalent and trivalent cations of the same metal is a major characterisation drawback for monometallic LDHs. Consequently, bimetallic and trimetallic LDHs have become the most common in many applications [26,27].

Given that the monometallic LDHs warrant further investigations, in this work we theoretically quantify the critical role of intercalant anions on the stability of three transition metal (TM) based LDHs, i.e., Mn, Fe, and Co, in monometallic form. We systematically investigate the role of the neutral water molecule, a single negatively charged lactate radical, and a double negatively charged carbonate radical as intercalants in these LDHs. Moreover, we also study the effect of intercalant anions on the bandgap of LDHs, which determines their optical properties. The latter part can aid in designing LDH-based photocatalysts, electrocatalysts, and photovoltaics.

## 2. Settings and Models

We performed spin-polarized first-principles density functional theory (DFT) calculations [28,29] using the CASTEP program [30]. The electron exchange-correlation energy was approximated with the local density approximation (LDA) within the Ceperley and Alder parametrisation (also known as CA–PZ) [31]. On-the-fly generated ultrasoft plane-wave pseudopotentials [32] were used to treat the core electrons. Dispersion effects were included using the semi-empirical dispersion correction based on the Ortmann–Bechstedt–Schmidt formalism [33,34]. The cut-off energy was set to 630 eV for all simulations. A tight Monkhorst–Pack *k*-point grid with 0.07 Å^−1^ spacing was used for integration over the Brillouin zone throughout all geometry optimisations. This spacing produced a 5×5×5 grid for un-intercalated LDH compounds. The density of states was calculated with a 10-times-denser grid. During the geometry optimisation [35], performed with fixed basis quality, internal coordinates and lattice parameters were relaxed to forces smaller than 0.01 eV Å^−1^ and energies smaller than 10^−5^ eV. When a supercell contained more than one magnetic ion, both ferromagnetic and antiferromagnetic spin alignments were examined, and the lowest energy configuration was used. Using true magnetic ground state is critical for obtaining realistic geometries and total energies [36].

We used an ad hoc Hubbard (*U*) term for the 3d electrons to correct the inherent and superficial electronic delocalisation associated with the LDA functional [37]. The LDA+U method with well-calibrated *U* values has been demonstrated to offer excellent electronic structures at a reasonable computational cost [38]. The values were 3.90 eV for the Mn 3d electrons, 5.30 eV for the Fe 3d electrons, and 3.32 eV for the Co 3d electrons. Our choice was guided by the values reported in the Materials Project database [39] for compounds containing these transition metal (TM) ions with similar oxidation states.

**Figure 1:**
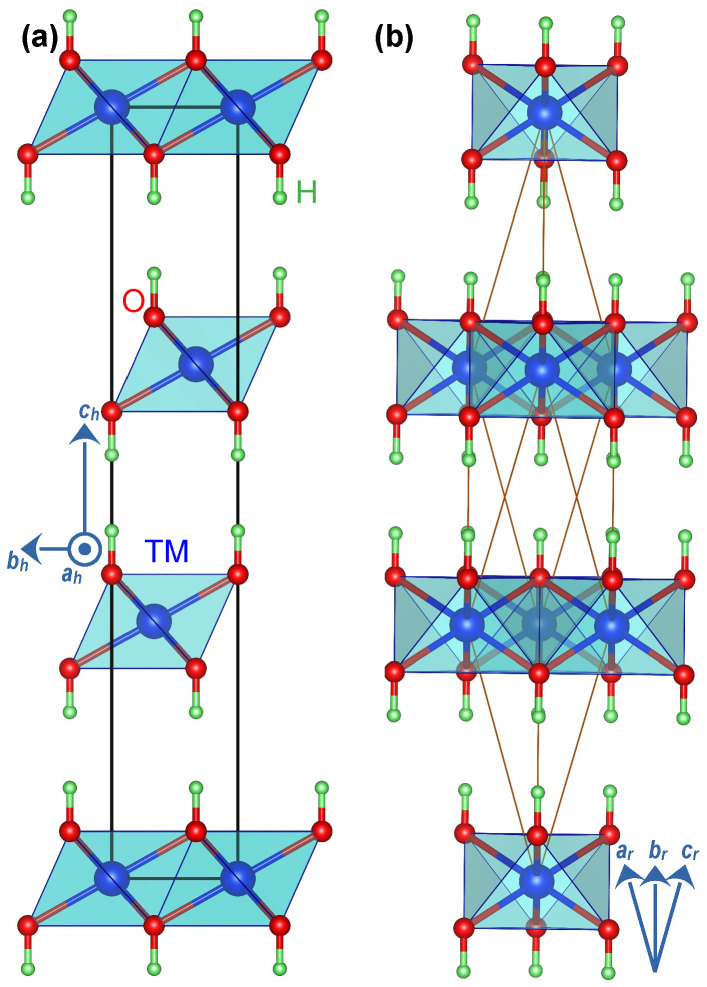
(**a**) The conventional cell of $TM{\left(OH\right)}_{2}$ layered double hydroxide compounds in hexagonal representation with group $R\bar{3}m$ and space group number 166. (**b**) The rhombohedral representation of the same structure used for most calculations. In hexagonal representation, $a_{h}=b_{h}$, $\alpha_{h}=\beta_{h}=90^{\circ }$, and $\gamma_{h}=120^{\circ }$. In the rhombohedral presentation, the lattice parameters $a_{r}=b_{r}=c_{r}$ and $\alpha_{r}=\beta_{r}=\gamma_{r}\neq 90^{\circ }$. The rhombohedral lattice parameters (denoted with subscript *r*) are related to hexagonal lattice parameters (denoted with subscript *h*) according to $\alpha_{r}=\arccos\left\{\left(2c_{h}^{2}-3a_{h}^{2}\right)/\left(2c_{h}^{2}+6a_{h}^{2}\right)\right\}$, and $a_{r}=\sqrt{\left(a_{h}^{2}/3\right)+\left(c_{h}^{2}/9\right)}$.

**Figure 2:**
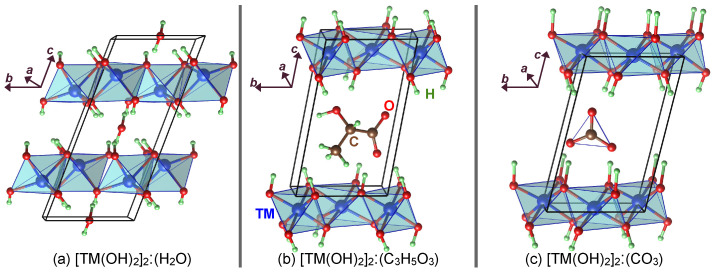
A representative of the LDH compounds intercalated with (**a**) $H_{2}O$, (**b**) $C_{3}H_{5}O_{3}$, and (**c**) $CO_{3}$. The optimised structures had higher cantered monoclinic symmetry with group C121 (group number 5) for the water intercalated compounds. The rest of the structures were P1.

We first optimised the un-intercalated structures. The un-intercalated compounds have a relatively simple hexagonal structure (Figure 1a) that has been widely reported in the literature. This hexagonal lattice has a primitive rhombohedral representation (Figure 1b) containing only one formula unit of $TM{\left(OH\right)}_{2}$ that simplifies the simulation. We then used the optimised un-intercalated structures to study the intercalated compounds. We constructed a 2a×1b×1c supercell from the primitive $TM{\left(OH\right)}_{2}$ unitcell and placed an intercalating species in the space between the $TM{\left(OH\right)}_{2}$ layers. We examined an exhaustive set of configurations for the intercalant’s possible spatial orientations and reported the most stable structures for each intercalating species, i.e., water molecule and lactate and carbonate radicals (Figure S1). We removed all symmetry constraints from the supercells during the geometry optimisation to allow for relaxation to lower symmetry structures should those yield lower energy. Finally, we probed the optimised structures, reported in Figure 2, for symmetry using the symmetry detection tool FINDSYM [40].

## 3. Results and Disscussion

We began with un-intercalated LDH compounds with an empty interlayer space. The geometry optimisation of the rhombohedral presentation, shown in Figure 1b, yielded a lattice parameter ar of 6.066 Å for $Mn{\left(OH\right)}_{2}$, 5.934 Å for $Fe{\left(OH\right)}_{2}$, and 5.841 Å for $Co{\left(OH\right)}_{2}$. Noticeably, all these structures were quite similar to one another. The optimised structures of the un-intercalated LDH compounds are presented in Table S1 of the supplementary materials. The Mn magnetisation based on the Mulliken population analysis was calculated to be 4.86 *μ_B_*, indicating a d^5^ electronic occupation, which corresponds to the +2 oxidation state and high spin configuration, arranged as filled spin-up $t_{2g}$ and $e_{g}$ orbitals and empty spin-down $t_{2g}$ and $e_{g}$ orbitals. The Fe magnetisation was calculated to be 3.80 $\mu_{B}$, which corresponds to the high spin configuration of d${}^{6}$ occupation ($t_{2g}^{3}$↑$e_{g}^{2}$↑$t_{2g}^{1}$↓$e_{g}^{0}$↓). The Co magnetisation was calculated to be 0.94 $\mu_{B}$, indicating that, unlike Mn and Fe, $Co^{2+}\left(d^{7}\right)$ is at a low-spin configuration of $t_{2g}^{3}$↑$t_{2g}^{3}$↓$e_{g}^{1}$↑$e_{g}^{0}$↓. One should note that the calculated magnetisations are slightly smaller than nominal values of pure ionic bonds—by a fraction of 1 $\mu_{B}$. This trend indicates that the TM-O bonds slightly deviate from pure ionicity towards covalency [41].

To examine the stability of these LDH compounds, we compared their DFT total energies against the most stable TM oxides by calculating the decomposition enthalpy (ΔH). These oxides were $Mn_{3}O_{4}$, $Fe_{2}O_{3}$, and $CoO_{2}$. The initial structures used to initiate the geometry optimisation of these oxides were taken from the Materials Project database [39]. The card numbers for $Mn_{3}O_{4}$, $Fe_{2}O_{3}$, and $CoO_{2}$ were mp-18759, mp-19770, and mvc-14149, respectively. We calculated (ΔH) per TM ion for these three LDH compounds according to the following equations:

(1) $$\Delta H=\left\{3E^{f}(Mn{\left(OH\right)}_{2})-E^{f}(Mn_{3}O_{4})-2E^{f}(H_{2}O)-E^{f}(H_{2})\right\}/3,$$

(2) $$\Delta H=\left\{2E^{f}(Fe{\left(OH\right)}_{2})-E^{f}(Fe_{2}O_{3})-E^{f}(H_{2}O)-E^{f}(H_{2})\right\}/2,$$

(3) $$\Delta H=\left\{2E^{f}(Co{\left(OH\right)}_{2})-2E^{f}(CoO_{2})-2E^{f}(H_{2})\right\}/2.$$

Here, $E^{f}$ is the DFT total energy of each compound.The ΔH was calculated to be −1.048 eV/Mn for $Mn{\left(OH\right)}_{2}$, −1.562 eV/Fe for $Fe{\left(OH\right)}_{2}$, and −2.009 eV/Co for $Co{\left(OH\right)}_{2}$. These negative ΔH values indicate the relative stability of these LDH compounds against their oxide forms. We can also infer a trend of higher stability for heavier TM-ion-based LDH compounds. Interestingly, when we repeated the same calculations without including the dispersion effects, ΔH was slightly higher, but negative nonetheless, at −0.866 eV/Mn, −1.367 eV/Fe, and −1.655 eV/Co, respectively. Higher but negative ΔH values show that although the dispersion effect contributes to the stability of these compounds, it is not the sole stabilising factor.

Figure 2 shows the optimised structures of the $H_{2}O$, $C_{3}H_{5}O_{3}$, and $CO_{3}$ intercalated LDH compounds. The relaxed lattice parameters and the atomic coordinates of all structures are provided in Table S2 and File S1 (in the common CAR format) of the supplementary information. For the water intercalated Mn LDH compound, the magnetisation of both Mn ions was 4.88 $\mu_{B}$, indicating that water intercalation did not change the Mn electronic configuration compared to the un-intercalated compound. For the lactate intercalated compounds, ${\left[Mn{\left(OH\right)}_{2}\right]}_{2}:C_{3}H_{5}O_{3}$, the magnetisation of one of the TM ions was reduced to 4.07 $\mu_{B}$, i.e., high-spin d${}^{4}$ configuration, indicating that the singly negative lactate radical oxidises one of the Mn ions to $Mn^{3+}$. In the case of doubly negative carbonate intercalation, both Mn ions had a magnetisation of 4.05 $\mu_{B}$, meaning both were at +3 oxidation state. To examine how intercalation affects stability, we calculated the decomposition enthalpy per Mn of the three intercalated Mn-based LDH compounds according to the following equations:

(4) $$\Delta H=\left\{3E^{f}{\left([Mn{\left(OH\right)}_{2}\right]}_{2}:H_{2}O)-2E^{f}(Mn_{3}O_{4})-7E^{f}(H_{2}O)-2E^{f}(H_{2})\right\}/6,$$

(5) $$\Delta H=\left\{3E^{f}{\left([Mn{\left(OH\right)}_{2}\right]}_{2}:C_{3}H_{5}O_{3})-2E^{f}(Mn_{3}O_{4})-3E^{f}(C_{3}H_{6}O_{3})-4E^{f}(H_{2}O)-0.5E^{f}(H_{2})\right\}/6,$$

(6) $$\Delta H=\left\{3E^{f}{\left([Mn{\left(OH\right)}_{2}\right]}_{2}:CO_{3})-2E^{f}(Mn_{3}O_{4})-3E^{f}(H_{2}CO_{3})-3E^{f}(H_{2}O)-0.5E^{f}(O_{2})\right\}/6.$$

We found ΔH was −2.271 eV/Mn for ${\left[Mn{\left(OH\right)}_{2}\right]}_{2}:H_{2}O$, −2.697 eV/Mn for ${\left[Mn{\left(OH\right)}_{2}\right]}_{2}:C_{3}H_{5}O_{3}$, and −1.735 eV/Mn for ${\left[Mn{\left(OH\right)}_{2}\right]}_{2}:CO_{3}$. These values are lower than the un-intercalated $Mn{\left(OH\right)}_{2}$ ( ΔH=−1.048 eV/Mn), therefore demonstrating higher stability. Consequently, intercalation enhances the stability of the Mn-based compounds. The greatest stability boost, however, is caused by lactate intercalation.

For the water intercalated Fe LDH, ${\left[Fe{\left(OH\right)}_{2}\right]}_{2}:H_{2}O$, the magnetisation of both Fe ions was found to be 3.82 $\mu_{B}$, indicating a high-spin d${}^{6}$ configuration—just like the un-intercalated compound. For lactate intercalation, ${\left[Fe{\left(OH\right)}_{2}\right]}_{2}:C_{3}H_{5}O_{3}$, the two Fe ions in the supercell had magnetisations of 3.86 $\mu_{B}$ and 4.20 $\mu_{B}$, indicating that the Fe ion with the larger magnetic moment was oxidised to $Fe^{3+}(d^{5})$. For carbonate intercalation, [Fe(OH)_2_]_2_:CO_3_, the magnetisation of both Fe ions was 4.21 $\mu_{B}$, indicating that both Fe ions were at +3 oxidation state. The stability trend was examined by calculating the decomposition enthalpy according to the following equations:

(7) $$\Delta H=\left\{E^{f}{\left([Fe{\left(OH\right)}_{2}\right]}_{2}:H_{2}O)-E^{f}(Fe_{2}O_{3})-2E^{f}(H_{2}O)-E^{f}(H_{2})\right\}/2,$$

(8) $$\Delta H=\left\{E^{f}{\left([Fe{\left(OH\right)}_{2}\right]}_{2}:C_{3}H_{5}O_{3})-E^{f}(Fe_{2}O_{3})-E^{f}(C_{3}H_{6}O_{3})-E^{f}(H_{2}O)-0.5E^{f}(H_{2})\right\}/2,$$

(9) $$\Delta H=\left\{E^{f}{\left([Fe{\left(OH\right)}_{2}\right]}_{2}:CO_{3})-E^{f}(Fe_{2}O_{3})-E^{f}(H_{2}CO_{3})-E^{f}(H_{2}O)\right\}/2.$$

We found ΔH was −1.456 eV/Fe for ${\left[Fe{\left(OH\right)}_{2}\right]}_{2}:H_{2}O$, −2.665 eV/Fe for ${\left[Fe{\left(OH\right)}_{2}\right]}_{2}:C_{3}H_{5}O_{3}$, and −1.318 eV/Fe for ${\left[Fe{\left(OH\right)}_{2}\right]}_{2}:CO_{3}$. Among these compounds, only ${\left[Fe{\left(OH\right)}_{2}\right]}_{2}:C_{3}H_{5}O_{3}$ is more stable than un-intercalated $Fe{\left(OH\right)}_{2}$, which had a ΔH value of −1.562 eV/Fe. Consequently, only lactate intercalation enhances the stability of the Fe LDH compounds. However, since ΔH remains negative for the latter intercalations, ${\left[Fe{\left(OH\right)}_{2}\right]}_{2}:H_{2}O$ and ${\left[Fe{\left(OH\right)}_{2}\right]}_{2}:CO_{3}$ are expected to survive equilibrium conditions.

Regarding the Co-based LDH compounds, we found that in the case of water intercalation, ${\left[Co{\left(OH\right)}_{2}\right]}_{2}:H_{2}O$, both Co ions in the supercell had a magnetisation of 0.95 $\mu_{B}$, corresponding with the low-spin d${}^{7}$ electronic configuration. For lactate intercalation in ${\left[Co{\left(OH\right)}_{2}\right]}_{2}:C_{3}H_{5}O_{3}$, the magnetisation of one Co ion remained 0.95 $\mu_{B}$, while the other Co became nonmagnetic. Nonmagnetic cobalt indicates a +3 oxidation state (d${}^{6}$), in which at low-spin configuration, the spin-up and spin-down electrons in the $t_{2g}$ orbitals cancel each other’s magnetisation. For the carbonate intercalated ${\left[Fe{\left(OH\right)}_{2}\right]}_{2}:CO_{3}$, both Co ions were nonmagnetic, indicating that the compound was comprised of $Co^{3+}$ only. The decomposition enthalpy for Co-based LDH compounds was calculated based on the following equations:

(10) $$\Delta H=\left\{E^{f}({\left[Co{\left(OH\right)}_{2}\right]}_{2}:H_{2}O)-2E^{f}(CoO_{2})-E^{f}(H_{2}O)-2E^{f}(H_{2})\right\}/2,$$

(11) $$\Delta H=\left\{E^{f}({\left[Co{\left(OH\right)}_{2}\right]}_{2}:C_{3}H_{5}O_{3})-2E^{f}(CoO_{2})-E^{f}(C_{3}H_{6}O_{3})-1.5E^{f}(H_{2})\right\}/2,$$

(12) $$\Delta H=\left\{E^{f}({\left[Co{\left(OH\right)}_{2}\right]}_{2}:CO_{3})-2E^{f}(CoO_{2})-E^{f}(H_{2}CO_{3})-E^{f}(H_{2})\right\}/2.$$

We found ΔH was −3.470 eV/Co for ${\left[Co{\left(OH\right)}_{2}\right]}_{2}:H_{2}O$, −3.968 eV/Co for ${\left[Co{\left(OH\right)}_{2}\right]}_{2}:C_{3}H_{5}O_{3}$, and −3.212 eV/Co for ${\left[Co{\left(OH\right)}_{2}\right]}_{2}:CO_{3}$. The values are substantially lower than the ΔH of the un-intercalated $Co{\left(OH\right)}_{2}$ of −2.009 eV/Co. Consequently, intercalating Co-based LDH with either a water, lactate, or carbonate molecule results in significant stabilisation. However, the stabilisation is greatest for lactate intercalation. Our prediction of stabilisation through lactate intercalation corroborates experimental observations in Fe- [19] and (Zn, Al)- [42] based LDH compounds.

**Figure 3:**
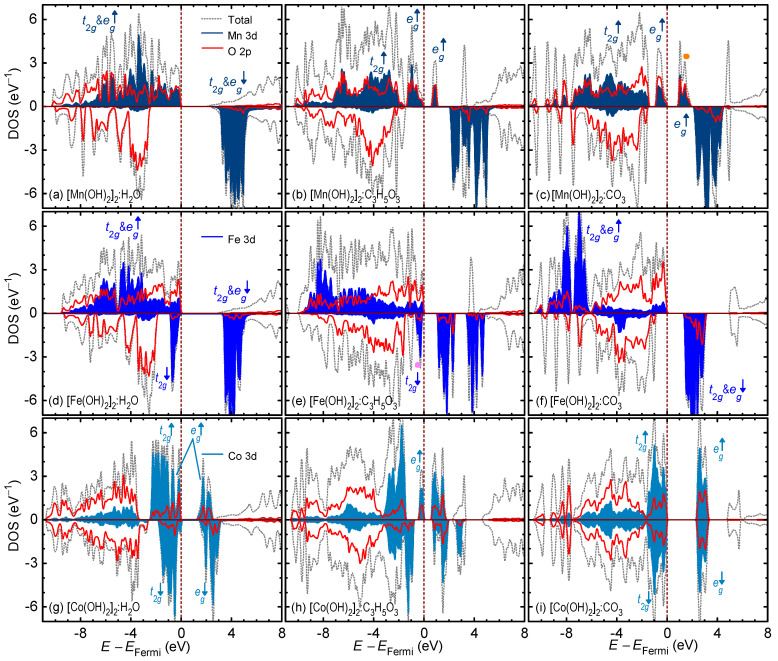
Total and partial density of state of the intercalated layered double hydroxide compounds. The upper row of (**a**–**c**) corresponds to the Mn-based compounds. The middle row of (**d**–**f**) corresponds to the Fe-based compounds. The lower row of (**g**–**i**) corresponds to the Co-based compounds. The first, second, and third columns correspond to $H_{2}O$, $C_{3}H_{5}O_{3}$, and $CO_{3}$ intercalation. The 3d partial density of states of different TM ions is shown in various shades of blue.

The total and partial density of states (DOS and PDOS) for all the intercalated compounds are shown in Figure 3. The 3d PDOS of all TM ions corroborate the arguments presented earlier from the TM magnetic moment viewpoint. Going from left to right— intercalation with water, then lactate, then carbonate—we can see the emptying of the d shell electrons due to oxidation, i.e., the states shifting to above the Fermi level. For Mn, that would be the emptying of spin-up $e_{g}$ states (marked with an orange circle). For Fe, that would be emptying the spin-down $t_{2g}$ states (marked with a purple circle). Lastly, for Co, that would be emptying the spin-up $e_{g}$ states. Figure 3 also show the bandgaps ($E_{g}$) for the intercalated LDH compounds. The $E_{g}$ value determines how responsive a compound is to photoexcitation. For instance, for electron–hole pair generation under visible light, an $E_{g}$ value of ∼1.7 eV is required. The $E_{g}$ for Mn-based LDHs was calculated to be 1.995 eV for ${\left[Mn{\left(OH\right)}_{2}\right]}_{2}:H_{2}O$, 0.480 eV for ${\left[Mn{\left(OH\right)}_{2}\right]}_{2}:C_{3}H_{5}O_{3}$, and 0.679 eV for ${\left[Mn{\left(OH\right)}_{2}\right]}_{2}:CO_{3}$. The ${\left[Mn{\left(OH\right)}_{2}\right]}_{2}:H_{2}O$ with half-filled d${}^{5}$ configurations had the widest bandgap because of the strong magnetic exchange between the spin-up and spin-down states. The $E_{g}$ for Fe-based LDHs was calculated to be 3.115 eV for ${\left[Fe{\left(OH\right)}_{2}\right]}_{2}:H_{2}O$, 1.027 eV for ${\left[Fe{\left(OH\right)}_{2}\right]}_{2}:C_{3}H_{5}O_{3}$, and 1.124 eV for ${\left[Fe{\left(OH\right)}_{2}\right]}_{2}:CO_{3}$. Finally, The $E_{g}$ for Co-based LDHs was calculated to be 1.055 eV for ${\left[Co{\left(OH\right)}_{2}\right]}_{2}:H_{2}O$, 0.307 eV for ${\left[Co{\left(OH\right)}_{2}\right]}_{2}:C_{3}H_{5}O_{3}$, and 2.047 eV for ${\left[Co{\left(OH\right)}_{2}\right]}_{2}:CO_{3}$. Here, the large crystal field splitting between the filled $t_{2g}$ states and the empty $e_{g}$ states widens the bandgap in ${\left[Co{\left(OH\right)}_{2}\right]}_{2}:CO_{3}$.

## 4. Conclusions

Using density functional calculations within the LDA + *U* formalism, we demonstrated that Mn-, Fe-, and Co-based layered double hydroxide compounds were stable against decomposition to the respective most stable oxides. Furthermore, in Mn- and Co-based LDH compounds, this stability is enhanced with either water, lactate, or carbonate intercalation. However, the most significant margin of stability was achieved for lactate intercalation. In Fe-based LDH compounds, water and carbonate intercalation reduced the margin of stability against decomposition to $Fe_{2}O_{3}$. In this case, only lactate intercalation improved the stability. Finally, we demonstrated that the intercalated LDHs have a large range of bandgaps, ranging from wide 3.115 eV in ${\left[Fe{\left(OH\right)}_{2}\right]}_{2}:H_{2}O$ to narrow 0.307 eV in ${\left[Co{\left(OH\right)}_{2}\right]}_{2}:C_{3}H_{5}O_{3}$. As a result, by controlling the intercalation molecule, one can tune the band gaps in these compounds for the desired applications.

## Data Availability

The data presented in this study are available in the Supplementary Materials.

## Supplementary Materials

The following supporting information can be downloaded at: https://www.mdpi.com/article/10.3390/nano12081339/s1, Figure S1: Search for the water intercalant orientation; File S1: The optimised structures for intercalated LDH compounds; Table S1: Lattice parameters of the un-intercalated LDHs; Table S2: The lattice parameters of the intercalated LDHs.

## References

[1] Doustkhah E., Ide Y. (2019). Bursting Exfoliation of a Microporous Layered Silicate to Three-Dimensionally Meso-Microporous Nanosheets for Improved Molecular Recognition. ACS Appl. Nano Mater..

[2] Doustkhah E., Ide Y. (2020). Microporous layered silicates: Old but new microporous materials. New J. Chem..

[3] Doustkhah E., Assadi M.H.N., Komaguchi K., Tsunoji N., Esmat M., Fukata N., Tomita O., Abe R., Ohtani B., Ide Y. (2021). In situ Blue titania via band shape engineering for exceptional solar H_2_ production in rutile TiO_2_. Appl. Catal. B.

[4] Wang Q., O’Hare D. (2012). Recent advances in the synthesis and application of layered double hydroxide (LDH) nanosheets. Chem. Rev..

[5] Huo W., Cao T., Liu X., Xu W., Dong B., Zhang Y., Dong F. (2019). Anion intercalated layered-double-hydroxide structure for efficient photocatalytic NO remove. Green Energy Environ..

[6] Wang Q., Tay H.H., Ng D.J.W., Chen L., Liu Y., Chang J., Zhong Z., Luo J., Borgna A. (2010). The Effect of Trivalent Cations on the Performance of Mg-M-CO_3_ Layered Double Hydroxides for High-Temperature CO_2_ Capture. ChemSusChem.

[7] Doustkhah E., Hassandoost R., Khataee A., Luque R., Assadi M.H.N. (2021). Hard-templated metal-organic frameworks for advanced applications. Chem. Soc. Rev..

[8] Mohapatra L., Parida K. (2016). A review on the recent progress, challenges and perspective of layered double hydroxides as promising photocatalysts. J. Mater. Chem. A.

[9] Cai X., Shen X., Ma L., Ji Z., Xu C., Yuan A. (2015). Solvothermal synthesis of NiCo-layered double hydroxide nanosheets decorated on RGO sheets for high performance supercapacitor. Chem. Eng. J..

[10] Balcik C., Ozbey-Unal B., Cifcioglu-Gozuacik B., Keyikoglu R., Karagunduz A., Khataee A. (2022). Fabrication of PSf nanocomposite membranes incorporated with ZnFe layered double hydroxide for separation and antifouling aspects. Sep. Purif. Technol..

[11] Li B., He J.G., Evans D., Duan X. (2004). Inorganic layered double hydroxides as a drug delivery system-intercalation and in vitro release of fenbufen. Appl. Clay Sci..

[12] Bi X., Zhang H., Dou L. (2014). Layered Double Hydroxide-Based Nanocarriers for Drug Delivery. Pharmaceutics.

[13] Constantino V.R., Pinnavaia T.J. (1995). Basic properties of ${\mathrm{Mg}}_{1-x}^{2+}{\mathrm{Al}}_{x}^{3+}$ layered double hydroxides intercalated by carbonate, hydroxide, chloride, and sulfate anions. Inorg. Chem..

[14] Lu Z., Zhu W., Lei X., Williams G.R., O’Hare D., Chang Z., Sun X., Duan X. (2012). High pseudocapacitive cobalt carbonate hydroxide films derived from CoAl layered double hydroxides. Nanoscale.

[15] Parida K., Mohapatra L. (2012). Carbonate intercalated Zn/Fe layered double hydroxide: A novel photocatalyst for the enhanced photo degradation of azo dyes. Chem. Eng. J..

[16] Okoronkwo M.U., Glasser F.P. (2016). Strätlingite: Compatibility with sulfate and carbonate cement phases. Mater. Struct..

[17] Sasai R., Sato H., Sugata M., Fujimura T., Ishihara S., Deguchi K., Ohki S., Tansho M., Shimizu T., Oita N. (2019). Why Do Carbonate Anions Have Extremely High Stability in the Interlayer Space of Layered Double Hydroxides? Case Study of Layered Double Hydroxide Consisting of Mg and Al (Mg/Al = 2). Inorg. Chem..

[18] Goh K.H., Lim T.T., Dong Z. (2009). Enhanced Arsenic Removal by Hydrothermally Treated Nanocrystalline Mg/Al Layered Double Hydroxide with Nitrate Intercalation. Environ. Sci. Technol..

[19] Tahawy R., Doustkhah E., Abdel-Aal E.S.A., Esmat M., Farghaly F.E., El-Hosainy H., Tsunoji N., El-Hosiny F.I., Yamauchi Y., Assadi M.H.N. (2021). Exceptionally stable green rust, a mixed-valent iron-layered double hydroxide, as an efficient solar photocatalyst for H_2_ production from ammonia borane. Appl. Catal. B.

[20] Mishra G., Dash B., Pandey S. (2018). Layered double hydroxides: A brief review from fundamentals to application as evolving biomaterials. Appl. Clay Sci..

[21] Mallakpour S., Hatami M., Hussain C.M. (2020). Recent innovations in functionalized layered double hydroxides: Fabrication, characterization, and industrial applications. Adv. Colloid Interface Sci..

[22] Palmer S.J., Frost R.L., Nguyen T. (2009). Hydrotalcites and their role in coordination of anions in Bayer liquors: Anion binding in layered double hydroxides. Coord. Chem. Rev..

[23] Bookin A., Drits V. (1993). Polytype diversity of the hydrotalcite-like minerals I. Possible polytypes and their diffraction features. Clays Clay Miner..

[24] Fan G., Li F., Evans D.G., Duan X. (2014). Catalytic applications of layered double hydroxides: Recent advances and perspectives. Chem. Soc. Rev..

[25] Dewangan N., Hui W.M., Jayaprakash S., Bawah A.R., Poerjoto A.J., Jie T., Jangam A., Hidajat K., Kawi S. (2020). Recent progress on layered double hydroxide (LDH) derived metal-based catalysts for CO_2_ conversion to valuable chemicals. Catal. Today.

[26] Wang Y., Yan D., El Hankari S., Zou Y., Wang S. (2018). Recent Progress on Layered Double Hydroxides and Their Derivatives for Electrocatalytic Water Splitting. Adv. Sci..

[27] Xu M., Wei M. (2018). Layered Double Hydroxide-Based Catalysts: Recent Advances in Preparation, Structure, and Applications. Adv. Funct. Mater..

[28] Kohn W., Sham L.J. (1965). Self-consistent equations including exchange and correlation effects. Phys. Rev..

[29] Payne M.C., Teter M.P., Allan D.C., Arias T., Joannopoulos J.D. (1992). Iterative minimization techniques for ab initio total-energy calculations - molecular-dynamics and conjugate gradients. Rev. Mod. Phys..

[30] Clark S.J., Segall M.D., Pickard C.J., Hasnip P.J., Probert M.I.J., Refson K., Payne M.C. (2005). First principles methods using CASTEP. Z. Kristallogr. Cryst. Mater..

[31] Ceperley D.M., Alder B.J. (1980). Ground State of the Electron Gas by a Stochastic Method. Phys. Rev. Lett..

[32] Lejaeghere K., Speybroeck V.V., Oost G.V., Cottenier S. (2014). Error Estimates for Solid-State Density-Functional Theory Predictions: An Overview by Means of the Ground-State Elemental Crystals. Crit. Rev. Solid State Mater. Sci..

[33] Ortmann F., Bechstedt F., Schmidt W.G. (2006). Semiempirical van der Waals correction to the density functional description of solids and molecular structures. Phys. Rev. B.

[34] McNellis E.R., Meyer J., Reuter K. (2009). Azobenzene at coinage metal surfaces: Role of dispersive van der Waals interactions. Phys. Rev. B.

[35] Pfrommer B.G., Cote M., Louie S.G., Cohen M.L. (1997). Relaxation of crystals with the quasi-Newton method. J. Comput. Phys..

[36] Pham A., Assadi M.H.N., Yu A.B., Li S. (2014). Critical role of Fock exchange in characterizing dopant geometry and magnetic interaction in magnetic semiconductors. Phys. Rev. B.

[37] Cococcioni M., de Gironcoli S. (2005). Linear response approach to the calculation of the effective interaction parameters in the *LDA*+*U* method. Phys. Rev. B.

[38] Loschen C., Carrasco J., Neyman K.M., Illas F. (2007). First-principles LDA + U and GGA + U study of cerium oxides: Dependence on the effective U parameter. Phys. Rev. B.

[39] Jain A., Ong S.P., Hautier G., Chen W., Richards W.D., Dacek S., Cholia S., Gunter D., Skinner D., Ceder G. (2013). The Materials Project: A materials genome approach to accelerating materials innovation. APL Mater..

[40] Stokes H.T., Hatch D.M. (2005). *FINDSYM*: Program for identifying the space-group symmetry of a crystal. J. Appl. Crystallogr..

[41] Assadi M.H.N., Katayama-Yoshida H. (2019). Covalency a Pathway for Achieving High Magnetisation in TMFe_2_O_4_ Compounds. J. Phys. Soc. Jpn..

[42] Jaubertie C., Holgado M., San Román M., Rives V. (2006). Structural characterization and delamination of lactate-intercalated Zn, Al-layered double hydroxides. Chem. Mater..

