# Supplementary material for: Stability Trends in Mono-Metallic 3d Layered Double Hydroxides
Source: Nanomaterials (Basel). 2022 Apr 13;12(8):1339. doi: 10.3390/nano12081339 (PMC9029406; doi:10.3390/nano12081339)
Supplement: Supplementary file 1 [file nanomaterials-12-01339-s001.zip › SupplementaryInfo.pdf]

Stability Trends in Mono-metallic 3d Layered Double Hydroxides

Saeedeh Mohammadi,[1] Ayoub Esmailpour,[1] Esmail Doustkhah,[2]* M. Hussein N. Assadi[3]*

[1]*Department of Physics, Shahid Rajaee Teacher Training University, Lavizan, Tehran 16788–15811, Iran.* s.mohammadi@sru.ac.ir (S.M.); esmailpour@sru.ac.ir (A.E.)

[2]*Koç University Tüpraş Energy Center (KUTEM), Department of Chemistry, Koç University, 34450 Istanbul, Turkey.* edoustkhahheragh@ku.edu.tr

[3]*RIKEN Center for Emergent Matter Science (CEMS), 2-1 Hirosawa, Wako, Saitama 351–0198, Japan.* mohammad.alassadi@riken.jp

*Correspondence: edoustkhahheragh@ku.edu.tr, mohammad.alassadi@riken.jp

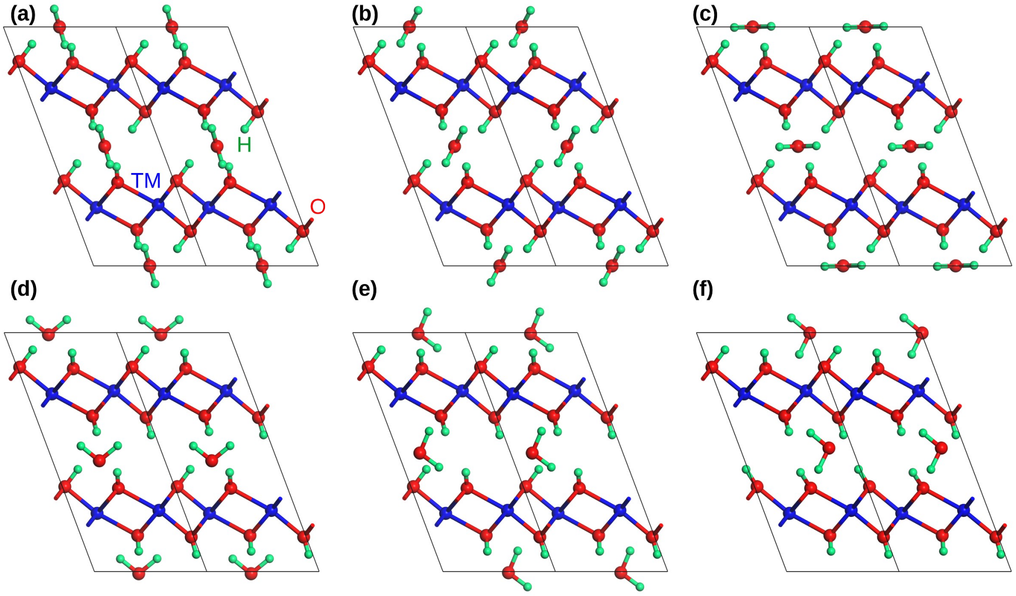

Figure S1. Initial H_2O molecule configurations used to identify the most stable water intercalated LDH compounds. These initial structures were created by fixing the relative atomic positions within the H_2O molecule and imposing a minimum 1.0 Å distance between the H_2O molecule atoms and the $TM(OH)_2$ layers, searching within 5° and 0.2 Å increments. The same procedure was repeated for lactate and carbonate intercalants.

Table S1. The lattice parameters of the un-intercalated layered double hydroxides $TM(OH)_2$ in both rhombohedral and hexagonal representations. The rhombohedral parameters are marked with subscript r, while the hexagonal parameters are marked with subscript h. The rhombohedral presentation contains one formula unit while the hexagonal presentation contains three formula units.

	a_r (Å)	α_r (°)	a_h (Å)	c_h (Å)
$Mn(OH)_2$	6.066	31.21	3.264	17.299
$Fe(OH)_2$	5.934	31.28	3.199	16.916
$Co(OH)_2$	5.841	29.39	2.964	16.756

Table S2. The lattice parameters of the water, lactate, and carbonate intercalated layered double hydroxides. The lattice parameters are presented for the primitive cells. The structure files of these compounds are reported in File S1.

	a (Å)	b (Å)	c (Å)	α (°)	β (°)	γ (°)
$[Mn(OH)_2]_2$:H_2O	6.132	6.132	5.253	110.13	110.13	32.73
$[Mn(OH)_2]_2$:$C_3H_5O_3$	3.257	6.138	9.093	75.03	80.29	58.69
$[Mn(OH)_2]_2$:CO_3	3.107	5.139	8.358	75.87	85.59	90.51
$[Fe(OH)_2]_2$:H_2O	6.132	6.132	5.253	110.13	110.13	32.73
$[Fe(OH)_2]_2$:$C_3H_5O_3$	3.203	5.792	9.048	76.57	80.74	65.50
$[Fe(OH)_2]_2$:CO_3	3.006	5.209	8.344	76.69	84.50	90.39
$[Co(OH)_2]_2$:H_2O	2.762	5.255	6.875	65.12	89.22	89.82
$[Co(OH)_2]_2$:$C_3H_5O_3$	3.024	5.027	9.406	74.97	100.65	87.24
$[Co(OH)_2]_2$:CO_3	2.816	4.844	8.149	78.06	84.70	90.28
